# Dysfunction in nonsense-mediated decay, protein homeostasis, mitochondrial function, and brain connectivity in ALS-FUS mice with cognitive deficits

**DOI:** 10.1186/s40478-020-01111-4

**Published:** 2021-01-06

**Authors:** Wan Yun Ho, Ira Agrawal, Sheue-Houy Tyan, Emma Sanford, Wei-Tang Chang, Kenneth Lim, Jolynn Ong, Bernice Siu Yan Tan, Aung Aung Kywe Moe, Regina Yu, Peiyan Wong, Greg Tucker-Kellogg, Edward Koo, Kai-Hsiang Chuang, Shuo-Chien Ling

**Affiliations:** 1 Department of Physiology, Yong Loo Lin School of Medicine, National University of Singapore, Singapore, 117549 Singapore; 2 Department of Medicine, Yong Loo Lin School of Medicine, National University of Singapore, Singapore, Singapore; 3 Agency for Science, Technology and Research, Singapore Bioimaging Consortium, Singapore, Singapore; 4 Computational Biology Programme, Faculty of Science, National University of Singapore, Singapore, Singapore; 5 Queensland Brain Institute, The University of Queensland, Brisbane, Australia; 6 Centre for Advanced Imaging, The University of Queensland, Brisbane, Australia; 7 Department of Pharmacology, Yong Loo Lin School of Medicine, National University of Singapore, Singapore, Singapore; 8 Program in Neuroscience and Behavior Disorders, Duke-NUS Medical School, Singapore, Singapore; 9 Department of Biological Sciences, Faculty of Science, National University of Singapore, Singapore, Singapore; 10 Department of Neurosciences, University of California at San Diego, La Jolla, USA; 11 Healthy Longevity Translational Research Programme, Yong Loo Lin School of Medicine, National University of Singapore, Singapore, Singapore; 12 Present Address: University of North Carolina, Chapel Hill, NC USA

**Keywords:** Amyotrophic lateral sclerosis (ALS), Frontotemporal dementia (FTD), FUS (fused in sarcoma), Auto-regulation, Nonsense-mediated decay (NMD), Protein homeostasis, Oxidation phosphorylation (OXPHOS), Brain connectivity, Functional magnetic resonance imaging (fMRI)

## Abstract

Amyotrophic lateral sclerosis (ALS) and frontotemporal dementia (FTD) represent two ends of the same disease spectrum of adult-onset neurodegenerative diseases that affect the motor and cognitive functions, respectively. Multiple common genetic loci such as fused in sarcoma (FUS) have been identified to play a role in ALS and FTD etiology. Current studies indicate that FUS mutations incur gain-of-toxic functions to drive ALS pathogenesis. However, how the disease-linked mutations of FUS affect cognition remains elusive. Using a mouse model expressing an ALS-linked human FUS mutation (R514G-FUS) that mimics endogenous expression patterns, we found that FUS proteins showed an age-dependent accumulation of FUS proteins despite the downregulation of mouse FUS mRNA by the R514G-FUS protein during aging. Furthermore, these mice developed cognitive deficits accompanied by a reduction in spine density and long-term potentiation (LTP) within the hippocampus. At the physiological expression level, mutant FUS is distributed in the nucleus and cytosol without apparent FUS aggregates or nuclear envelope defects. Unbiased transcriptomic analysis revealed a deregulation of genes that cluster in pathways involved in nonsense-mediated decay, protein homeostasis, and mitochondrial functions. Furthermore, the use of in vivo functional imaging demonstrated widespread reduction in cortical volumes but enhanced functional connectivity between hippocampus, basal ganglia and neocortex in R514G-FUS mice. Hence, our findings suggest that disease-linked mutation in FUS may lead to changes in proteostasis and mitochondrial dysfunction that in turn affect brain structure and connectivity resulting in cognitive deficits.

## Introduction

Since 2009, more than 60 mutations in fused in sarcoma (FUS), also known as translocated in liposarcoma (TLS), have been identified to be associated with the two overlapping adult-onset neurodegenerative diseases: amyotrophic lateral sclerosis (ALS) and frontotemporal dementia (FTD) [1, 2]. Although pathological FUS aggregates were originally found in ALS patients with FUS mutations [3, 4], abnormal FUS inclusions and increased cytosolic FUS have also been found in sporadic ALS patients [5, 6] and a subgroup of FTD patients without FUS mutations [7, 8]. This genetic and pathological data indicate that FUS dysfunction may be a common convergent pathway that leads to ALS and FTD. Furthermore, FTD patients with FUS inclusions belong to a group of atypical FTD patients that encompasses heterogeneous symptoms and widespread pathology throughout the central nervous system (CNS), including the hippocampus [7, 8]. While it is clear that there are deficits in executive functions that are due to the degeneration of the frontal cortex in the frontotemporal lobar degeneration (FTLD)-FUS patients [8], it is still unclear whether FUS pathology within hippocampus cause defects in hippocampus-dependent cognition.

Molecularly, FUS contains an amino-terminal prion-like, low complexity domain [9, 10], followed by a nuclear export signal, an RNA recognition motif domain, arginine/glycine-rich domains, a zinc-finger motif and a non-canonical proline-tyrosine nuclear localization signal (PY-NLS) at the extreme carboxyl terminus [11]. FUS binds to single- and double-stranded DNA as well as RNA, and participates in the regulation of gene expression, ranging from transcription, and post-transcriptional regulation, to mRNA localization and translation [2, 12–14]. FUS directly associates with RNA polymerase II (RNAP II) at the promoter region [15, 16] and is critical for the directionality of transcription [17]. In addition, FUS is co-transcriptionally deposited onto the newly synthesized pre-mRNA [18, 19], and it is also involved in transcription-splicing coupling, alternative splicing and polyadenylation site selection [20–23]. FUS shuttles between nucleus and cytosol [24] and forms ribonucleoprotein (RNP) granules involved in RNA localization and localized translation [25–27]. Moreover, FUS has been shown to take part in transporting RNA granules in dendrites and also to regulate dendritic and axonal translation [25, 28, 29]. In particular, FUS has been shown to regulate the expression of synaptic proteins by binding to the 3′-UTR of the GluR1 subunit of the α-amino-3-hydroxy-5-methyl-4-isoxazolepropionic acid (AMPA) receptors (encoded by *Gria1*) [30] and α2 isoform of synaptic Ras GTPase-activating protein 1 (SynGAP1) (encoded by *Syngap1*) [31], and transient FUS knockdown in hippocampus leads to behavioral abnormality related to FTD [30]. Taken together, FUS not only participates in biogenesis and processing of nuclear mRNAs, but it is also involved in dendritic and axonal mRNA transport and localized translation, that are important for regulating synaptic plasticity and integrity.

The prevalent FUS mutations that are causal for ALS are clustered at the C-terminal nuclear localization signal (NLS); they cause a shift of steady-state FUS localization from nucleus to cytosol. Furthermore, the severity of disease onset and progression correlates positively with the higher degree of cytoplasmic localized FUS [11, 14, 32, 33]. These observations led to the hypothesis that the loss of nuclear FUS functions, or gain of additional toxic properties, or both, may contribute to FUS toxicity [13, 34, 35]. FUS knockout mice do not develop ALS-like symptoms or pathology [36–38], suggesting that loss of FUS function is not sufficient to cause motor neuron diseases. Recent studies using human induced pluripotent stem cells (iPSCs)-derived motor neurons carrying FUS mutations [39–41], and various mouse models [28, 29, 34, 37, 38, 42, 43], indicate disease-linked FUS mutations use a “gain of toxicity” mechanism to drive ALS pathogenesis. It is worth mentioning that partial loss of FUS functions such as RNA misprocessing [23, 34, 44], dysfunctional paraspeckles [45], DNA damage repair [43, 46, 47], mitochondrial dysfunctions [48] and axonal translation [29], are also found in these cells and mouse models. Thus, it is likely that a combination of gain of toxic properties and loss of normal FUS functions contribute to the motor and cognitive deficits found in ALS and FTD patients.

It remains enigmatic why mutations in FUS preferentially provoke ALS-FTD spectrum disease and if these disease-causing FUS mutations also damage the hippocampus where FUS pathology is prevalent in FTLD-FUS patients. Herein, we combined biochemical, electrophysiological, immunohistological, transcriptomic, behavioral approaches and in vivo brain imaging to assess the hippocampal functions in a mouse model where an ALS-linked human FUS mutation (R514G-FUS) transgene was expressed in the CNS using a murine prion promoter [34]. Our analyses revealed wide range of molecular, cellular, physiological, and structural changes correlating with hippocampus-dependent cognitive deficits in R514G-FUS mice. Specifically, our data suggest that disease-linked mutation in FUS may lead to change in nonsense-mediated decay (NMD), proteostasis and mitochondrial functions, which in turn affect brain structure and connectivity resulting in cognitive deficits.

## Materials and methods

### Mouse models

All studies were carried out using protocols approved by the Institutional Animal Care and Use Committee from the National University of Singapore and were in compliance with Association for Assessment of Laboratory Animal Care guidelines for animal use. All mice used in this study were from the C57BL/6J strain and were housed in ventilated cages under a 12 light/12 dark cycle with access to food and water ad libitum. These WT-FUS and R514G mice developed lower motor neuron degeneration and motor phenotype without FUS aggregates as described previously [34]. As the R514G-FUS transgene is on X chromosome, only male mice were included and randomly allocated to experimental groups according to age and genotype. No other animals or samples were excluded in any of the experiments. For genotyping, genomic DNA was isolated from tail biopsies using salt extraction methods and subjected to routine PCR methods using the following primers: FUS transgene: 5′-gaggatttcccagtggaggt-3′ and 5′- ctccatcaaagggacctgaa-3′.

### Immunofluorescence

The preparation of tissues for immunofluorescence was described previously [34, 49]. Briefly, isoflurane was used to anesthetize the mice before they were transcardially perfused with phosphate buffered saline (PBS), and fixed with 4% paraformaldehyde (FPA) in phosphate buffer. Brain, spinal cord and gastrocnemius muscles were dissected and post-fixed in 4% PFA in PBS for 2 h. Cryopreservation of tissues in 30% sucrose over 24 h occurred before tissues were embedded in Tissue-Tek ready for sectioning. Brains were sectioned coronally at 30 μm using a cryostat or microtome and placed in PBS.

Antigen retrieval was done for all immunofluorescence analyses. Antigen retrieval was done via heating the tissues in Tri-Sodium citrate (10 mM sodium citrate, 0.05% Tween 20, pH 6.0) at 95 °C for 15 min prior to immunofluorescence staining. Brain tissue sections underwent three washes with 1X PBS and were treated with 10 mM glycine for 15 min each to remove any trace of PFA. The tissues were then permeabilized with 0.3% Triton X-100 in 1X PBS, and blocked in blocking serum, 5% donkey serum with 0.3% Triton X-100 in 1X PBS. Incubation with primary antibodies diluted in 1% donkey serum and 1X PBS in blocking serum were performed overnight at 4 °C. Tissues were then washed 3 times for 15 min in 1X PBS. Incubation of secondary antibodies conjugated with Alexa Fluor™ 488, 568, 643 (1:1000, Thermo Fisher Scientific) and 1 μg/ml DAPI staining was done in 0.1% Triton X-100 with 1% Donkey Serum in 1X PBS for overnight at 4 °C. Tissues were washed in 0.1% Triton X-100 in 1X PBS 3 times for 15 min before mounting them onto slides with Prolong Gold anti-fade reagent (Thermo Fisher Scientific, P36930). The primary antibodies used in this study were: mouse monoclonal FUS (1:200, Santa Cruz Biotechnology, clone 4H11), in-house rabbit polyclonal FUS (14082, in-house [34]; 1:400), rabbit polyclonal NeuN (D3S3I) (1:1000, Cell Signaling Technology, 12943S), HA-Tag (Bethyl Laboratories, A190-138A, 1:400), and RanGAP-1 (Thermo Fisher Scientific, 33-0800, 1:500).

### Tissue protein extraction and immunoblotting

Tissues were harvested and snap frozen in liquid nitrogen before protein was extracted. Total proteins (and RNA) was extracted from the hippocampi of non-transgenic and R514G mice using TRIzol reagent (Thermo Fisher Scientific) according to manufacturer’s instruction [50]. Briefly, tissue was homogenized in the TRIzol reagent using a homogenizer. The homogenate was subsequently mixed with chloroform and centrifuged. This yielded a top aqueous phase containing the RNA, an interphase containing the DNA, and the bottom organic phase that contained the protein. The aqueous phase was removed and the RNA precipitated with isopropanol (refer to RNA isolation section below). Ethanol was added to the remaining interphase and organic phases to precipitate the DNA. After centrifugation, the organic phenol-ethanol phase was transferred to a clean tube and precipitated in isopropanol. The supernatant was removed after centrifugation and the protein pellet was washed with 0.3 M guanidine hydrochloride in 95% ethanol. The protein pellet was briefly air-dried before being dissolved in 1% sodium dodecyl sulfate (SDS) and protein concentration determined using the Pierce™ BCA protein assay kit (Thermo Fisher Scientific, 23225). Thirty μg of total protein for each sample was used to perform protein gel electrophoresis using 10% Bis–Tris gels. Proteins were transferred to nitrocellulose membrane using 1x transfer buffer containing 1x Tris–Glycine (25 mM Tris base and 192 mM glycine) and 20% methanol supplemented with 0.02% SDS at 80 V for 120 min. Membranes were blocked using 5% milk in 1x TBST (50 mM Tris, 150 mM NaCl, 0.1% Tween 20, pH 7.4) at room temperature for 1 h. Membranes were subsequently incubated with primary antibodies at 4 °C overnight. The primary antibodies used in this study were: mouse monoclonal anti-FUS (Santa Cruz Biotechnology, clone 4H11, 1:500), and mouse anti-beta-tubulin (Developmental Studies Hybridoma Bank, clone E7, 1:10,000). After the primary antibody incubation, membranes were washed with 1X TBST and incubated with anti-mouse HRP-conjugated secondary antibodies (1:5000, Cell Signaling Technology, Inc) at room temperature for 1 h. Membranes were then washed extensively with1xTBST, before the target proteins were probed using SuperSignal™ West Pico Chemiluminescent Substrate (Thermo Fisher Scientific). Signals were acquired using ChemiDoc XRC+ system (Bio-Rad). Image Lab software (Bio-Rad) was used for quantification.

### Image acquisition

Confocal images were acquired using a Zeiss LSM700 inverted confocal microscope with 4 laser lines (405/488/555/639 nm), and either a 20x/0.8 N.A. air or 63x/1.15 N.A. oil immersion objectives. Images were captured using an AxioCam MRm monochromatic CCD camera (Zeiss) run by Zeiss Zen software.

### Acute hippocampal slice electrophysiology

Acute horizontal hippocampal slices were used to assess the extracellular field potential recordings of 6 month and 12 month-old non-transgenic and R514G mice. Measurements were taken from 5 to 7 mice for each cohort (genotype and age) with a minimum of 10 slices measured. On each experimental day only two mice, one R514G and one non-transgenic mouse, were sacrificed for analysis. The experimenter was blinded to the genotype of the mouse. Isoflurane was used to euthanasia the animals before the hippocampi’s were removed. Hippocampal slices were cut at 400-µm thick with a vibroslicer (Campden Instruments, Leicester, UK). Slices were transferred to a recording chamber in artificial cerebral spinal fluid (ACSF) containing (in mM) 125 NaCl, 2.4 KCl, 1.2 NaH_2_PO_4_, 1 CaCl_2_, 2 MgCl_2_, 25 NaHCO_3_, and 25 glucose and perfused with oxygenated ASCF containing 2 mM CaCl_2_ and 1 mM MgCl_2_. Using microelectrodes filled with extracellular recording solution, extracellular recordings of field excitatory postsynaptic potentials (fEPSPs) were obtained from the stratum radiatum in the CA1 area as described previously [49, 51]. Synaptic plasticity was assessed by the stimulation of the Schaffer collateral/commissural pathway by placing a bipolar stimulating electrode in the stratum radiatum in CA1 region. By comparing the input and output relationship of the fEPSPs recorded the basal synaptic transmission could be assessed. The fiber volley amplitude and initial slope of the fEPSP responses to a range of stimulation from 100 to 900 µA was measured for each animal. Once the maximal response (submaximal) was determined the stimulus intensity was adjusted to 30–40% for each individual mouse. By using the peak amplitude of the fiber volley (input) and the initial slope of the fEPSP (output) the strength of the synaptic transmission could be quantified. The long-term synaptic modification potential was measured by the long-term potentiation (LTP) that was induced by four tetani after a 20 min baseline period. The four tetani were delivered 20 s apart, each at 100 Hz for 1 s. The pClamp 10 software (Molecular Devices, Sunnyvale, CA, USA) was used to analyze the raw data.

### Golgi-Silver staining and spine quantification

Golgi-Cox impregnation was used to visualize the neurons and especially their dendritic spines. The FD Rapid GolgiStain kit was used according to the manufacturer’s instruction (FD NeuroTechnology, Cat# PK401) [49]. For quantitative analyses of the dendritic spines, the region of the apical dendrites of CA1 pyramidal neurons after the first branch point was selected (secondary dendrite). Neurons were randomly selected and three to five randomly chosen areas per neuron were selected for scoring on approximately three to five different neurons. Z-sections were taken at 0.3-μm intervals. The spine densities were calculated by dividing the number of spines in the chosen area by the length of dendrite of that area. All data were represented as mean ± SEM. A total of three biological replicates per genotype were used for the Golgi-Cox spine density analysis.

### Behavioral testing

The behavioral tests were performed as previously described [49, 50] and detailed below. As the progressive decline of motor and cognitive functionalities are hallmarks of neurodegenerative diseases, we reasoned that the longitudinal testing of the same animal may capture the disease progression more faithfully. Furthermore, if the assays are sensitive and the phenotype is strong, the progression of the phenotype may also be captured using a longitudinal design, and at the same time may negate the possibility of batch effects. In this study, the same animals were used in both 6- and 12-month time points with their littermate controls.

#### Open field test

Exploratory activity was assessed by placing animals in a square open field (25 × 25 cm) in a plexiglass cage for an hour. The behaviors were recorded and analyzed using the Topscan software (Cleversys Inc, Reston VA).

#### Novel object recognition test

Recognition memory was assessed using the novel object recognition test paradigm. Animals were exposed to the testing arena for two consecutive days. On the third day, animals were presented with two training objects for 10 min. Probe tests were carried out 20 min, 16–24 h and 10–14 days after the completion of training, for short-term (STM), long-term (LTM) and remote (RM) memory respectively. For probe tests, animals were returned to the arena for 10 min, where they were presented with one training object and one novel object. The sets of objects used were different for each of the assessed ages. Video recordings of the interactions with the objects were taken and analyzed by Topscan (Cleversys Inc, Reston VA). The data are presented as the interaction time that was spent with the novel object over total interaction time.

#### Morris water maze

The Morris water maze (MWM) was used as a test for spatial learning and memory as described previously [52]. MWM is an open circular pool (120 cm in diameter × 60 cm in height) filled with water (25 ± 1 °C) to a depth of 50 cm and rendered opaque by adding non-toxic poster paint. Four points around the tank were designated as North (N), South (S), East (E) or West (W). The tank area was equally divided into four quadrants (NW, SW, NE and SE). In this hidden, fixed platform paradigm, a circular Plexiglass platform (9 cm in diameter) was located at the center of the SE quadrant. The height of the platform was adjustable and submerged 1.5 cm below the water surface. Distal cues surrounding the tank were provided to the mice to help them navigate to the hidden platform. These cues were different for each of the ages assessed. Animals were trained for four trials per day for 5 days. In each trial, the mice were given 60 s to find the submerged platform. The time taken from when the animal was placed in the water until it reached the platform was measured as their escaped latency. If a mouse failed to find the platform, it was guided to the platform and allowed to remain there for 10 s, before being returned to its holding cage until the next trial was initiated. The mice were probed to test for memory retention 20 min after the training was completed on the first day for STM, and 24 h after the last training day for LTM. The test was recorded and analyzed by EthoVision XT (Noldus Information Technology, The Netherlands).

#### Contextual fear conditioning

Animals were placed into an operant chamber with a metal grid for foot shock application. Two minutes later, three electrical foot shocks were presented (0.55 mA, 2 s) with intershock interval of 1 min. Animals were returned to their home cages 2 min after the last foot shock. To test for STM, LTM and RM of contextual fear, the mice were placed back into the same operant chamber for 20 min, 16–24 h and 10–14 days after training, respectively. The movements of the mice in the chamber were recorded for 3 min using FreezeScan (Cleversys, Reston, VA, USA). The freezing response in the conditioning context was recorded as a measure of contextual fear memory. The contextual cues used were different for each age group.

### Statistical analysis

Data were expressed as mean ± SEM, and *p *< 0.05 was considered as statistically significant. The data were analysed using the R statistical program (R Foundation for Statistical Computing, Vienna, Austria). All data were assessed for normality of residuals and homogeneity of variance using the tools for building OLS regression and the Levene’s test from the car statistical package respectively. No violations to assumptions of normality and homogeneity of variation were found. The options{base} package was used to define the contrasts used to compute the parameters of the Anova. Pairwise t-tests with Bonferroni corrections were obtained using the with{base} package.

### RNA-Seq library preparation and sequencing, and bioinformatics analysis

#### Abundance quantification and differential expression calling

The Agilent Bioanalyzer system was used to measure the RNA quality before commencing the library preparation. A RNA integrity number (RIN) of larger than 8.0 was used as the quality assurance for the RNA library preparation samples. Multiplex strand specific RNA-seq libraries were prepared from 12-month-old mouse hippocampus RNA using Illumina TruSeq RNA Sample Prep Kit and libraries were sequenced using Illumina HiSeq 4000 single-ended 50 bp sequencing. Read quantification was performed with Kallisto (0.44.0) [53] with parameters -b 50 –single -l 200 -s 20 using ENSEMBL cDNA transcripts (release 91). Downstream differential gene expression calling was performed using Sleuth (0.28.1) [54]. Quantified genes from each sample were annotated with a condition tag corresponding to the sample genotype. For each gene, Wald testing was performed on the condition parameter to obtain their respective FDR-corrected p-values. Significance for each gene was then established under a cutoff of FDR < 0.1. RNA-seq data have been deposited in NCBI’s Gene Expression Omnibus with the GEO series accession number GSE157713. (https://www.ncbi.nlm.nih.gov/geo/query/acc.cgi?acc=GSE157713).

#### Functional analysis and generation of diagnostic and expression plots

Diagnostic plots (MA, Principal Components Analysis) were generated using the R statistical language’s ggplot2 package. Principal Components analysis was then performed on the batch-corrected gene Transcripts Per Million (TPM) values generated from Kallisto-sleuth gene expression quantifications. Heatmaps were generated using log-scaled Kallisto read counts. The expression matrix was then Z-scaled and centered before clustering across both rows and columns and rendered with the superheat R library [55]. Expression plots for differentially expressed genes were generated from control-normalized Kallisto TPM estimates. The Gene Ontology analysis and gene-set enrichment analysis using the Reactome database was done using the CLUEGO plugin [56] in Cytoscape [57]. All heatmaps were generated with the R package, superheat [55].

### RNA isolation and qRT-PCR

TRIzol™ reagent (Thermo Fisher Scientific) was used to extract total RNA from mouse hippocampi according to the manufacturer’s instruction. The total RNA then underwent a DNase treatment using RQ1 RNase-Free DNase (Promega), before the Maxima First Strand cDNA Synthesis Kit for RT-qPCR (Thermo Fisher Scientific) was used to reverse transcribe 1 µg of RNA. A minimum of at least three biological replicates for each group and three technical replicates were used for all qRT-PCR reactions. The Maxima SYBR Green qPCR master mix (Thermo Fisher Scientific) was used to determine the mRNA expression levels. Primer sequences are listed in Additional file 1: Table S1 and at least two reference genes were used for normalization (ARHGDIA, HPRT, and GAPDH). qRT-PCR results are displayed as relative mRNA expression levels normalized to the geometric mean of the reference genes.

### MRI data acquisition

#### Ex-vivo

MRI of the mouse brain samples (n = 5 for both genotypes) were acquired using a micro-2.5 gradient coil with 20 mm SAW volume coil (M2M Imaging, Brisbane, Australia) on a 16.4T MRI with 89 mm vertical bore magnet (Bruker BioSpin, Karlsruhe, Germany). After extraction from the skull, the brains were incubated in PBS for 2 days before incubation in 0.2% Gd-DTPA (Magnevist^®^, Bayer) for 4 days at 4 °C on an orbital shaker. The samples were then placed in Fomblimrprior to MRI scan. For anatomical imaging, T1-weighted 3D gradient echo images were acquired with repetition time = 30 ms, echo time = 1.24 ms, flip angle = 18° and 100 micron isotropic spatial resolution.

Functional MRI images were acquired on a 9.4T MRI with 30 cm horizontal bore magnet (Bruker) with a 72 mm quadrature volume coil (Bruker) for transmission and a 4-channel mouse brain array coil (Bruker) as the receiver. Mice were prepared using established procedures [58, 59]. Mice were initially anesthetized with 2% isoflurane. A bolus of 0.15 mg/kg medetomidine (Dormitor^®^, Pfizer, USA) was administered intraperitoneally 15 min after induction, followed by continuous infusion of 0.3 mg/kg/h medetomidine 10 min later. Isoflurane was tapered down to 0.2–0.5% based on the breathing rate. The respiration was monitored (SA Instruments, Inc., New York, USA) and the rectal temperature was maintained at ~ 37 °C by a warm water circulation system. fMRI scans were acquired at approximately 45 min after the medetomidine bolus injection by a gradient-echo echo-planar imaging with TR = 1 s, TE = 15 ms, thickness = 0.5 mm, gap = 0 mm, matrix size = 64 × 64, FOV = 20 × 20 mm^2^ and repetition = 600 (10 min). To correct for the geometric distortion, a reversed phase scan was acquired. Several runs of forepaw stimulation fMRI were conducted before the resting-state fMRI scan to ensure optimal neurovascular coupling was maintained.

### Image analysis

MRI data processing and statistical analysis were conducted using FMRIB Software Library (FSL ver 5.0; https://www.fmrib.ox.ac.uk/fsl), Advanced Normalization Tools (ANTs; http://stnava.github.io/ANTs/), SPM (https://www.fil.ion.ucl.ac.uk/spm/), AFNI (ver 19.0.09; https://afni.nimh.nih.gov/) and in-house codes on Matlab (Mathworks, MA, USA).

### Morphometry

The structural MRI data were preprocessed with N4 for intensity non-uniformity correction, followed by PCNN3D for brain extraction [60]. The resulted brain masks were manually edited to further match the outline of brain tissue, if necessary. For brain morphometric analysis, a study-specific brain template was first created from all of the extracted brains. Co-registering to the Australian Mouse Brain Mapping Consortium (AMBMC; http://www.imaging.org.au/AMBMC/AMBMC) atlas using linear and nonlinear transformation by ANTs was first performed. The co-registered brain images were then averaged to form a study-specific brain template. This process was iterated four times to generate a final study-specific template with all images co-registered to this template. The volumetric change in each voxel was analyzed using tensor-based morphometry technique [61]. This method uses the estimated deformation when registering to the template to calculate the volume change and has been successfully applied to detect morphological changes in human and animal studies. The Jacobian determinants of the deformation field were obtained in each voxel to represent the volume change of that voxel. To estimate the regional volume change, the anatomical labels of the AMBMC atlas were transferred to the study-specific template using ANTs and the regional volume was calculated by summing the Jacobian determinants in a labeled region and multiplying by the voxel resolution. A between groups comparison was conducted on the Jacobian determinants maps using FSL randomise and statistical significance was determined by permutation test with family-wise error rate corrected. Regional and whole brain volumes were calculated based on the AMBMC atlas label and statistical significance was tested using t-test (Prism, GraphPad LLC).

#### Functional connectivity

Resting-state fMRI data were preprocessed by an optimized pipeline we developed as described previously [58, 62]. Briefly, motion correction was done by SPM. A mask of ventricle, muscle and skin was created using a region-growing function followed by manual editing to avoid partial volume effect. The principal components of the voxel time-courses in the ventricle, muscle and skin, the six motion parameters, and the top 10 voxels with the highest temporal variation were used as the nuisance regressors. To correct geometric distortions FSL TOPUP was applied. Afterward, the images were coregistered to the AMBMC template. The data were further bandpass filtered from 0.01 to 0.1 Hz, and spatially smoothed with 0.5 mm Gaussian kernel. Functional connectivity was calculated as the Pearson’s correlation between the averaged signal in the labeled brain areas that include 246 regions with 74, 34 and 15 areas in each hemisphere of the cortex, basal ganglia and hippocampus, respectively. The connectivity matrix was calculated as correlation between the mean time course of each region. The correlation value was converted to z-score using Fisher’s z-transformation. The connectivity values of repeated scans were averaged. A between groups comparison was conducted on the z-score map and matrix using t-test, with multiple comparisons controlled by false discovery rate at *p* < 0.01.

## Results

### Transgene expressing disease-linked mutation in FUS showed cytosolic distribution and less effective down-regulation of endogenous FUS

**Fig. 1:**
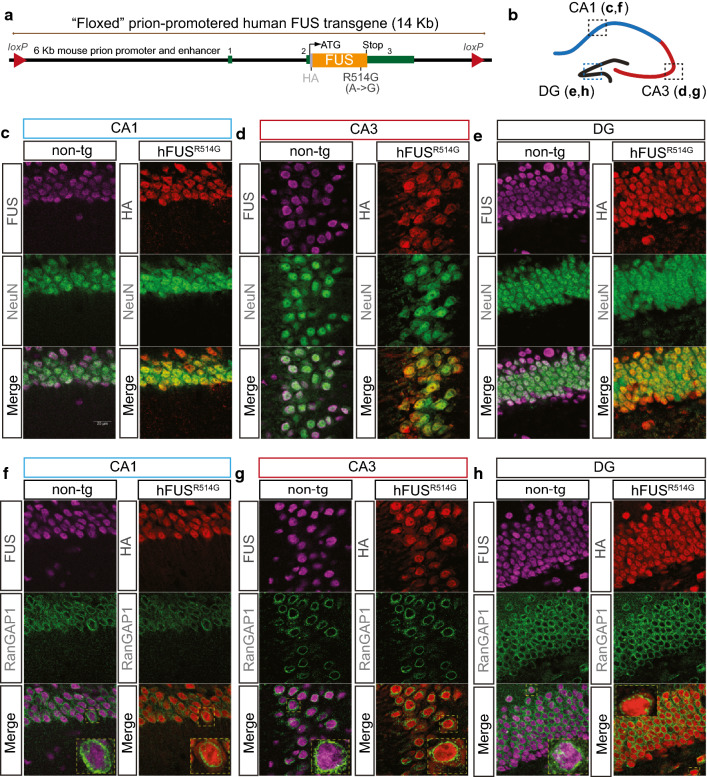
ALS-linked mutant FUS transgene is restricted to neurons and has cytosolic localization. **a** Schematic of FUS transgene. Murine prion promoter was used to drive the HA-tagged human FUS cDNA expression (R514G). **b** Schematic representation of different subregions (CA1, CA3 and DG) within hippocampus, and used for panels **c**–**h**. Confocal images of endogenous FUS (magenta) and human FUS transgene (red) in the CA1 (**c**), CA3 (**d**) and DG (**e**) of the hippocampus at 3 months of age. Transgenic FUS protein was detected using a HA antibody. Both endogenous FUS and human FUS transgene were present in the neuronal nucleus. Scale bar is 20 μm. Confocal images of the CA1 (**f**), CA3 (**g**), and DG (**h**) regions that were co-labeled with endogenous FUS (magenta) or R514G-FUS transgene (red) and nuclear envelope marker (green) from non-transgenic and R514G mice at 3 months of age. Endogenous FUS (magenta) is restricted to nuclei, whereas R514G-FUS transgene (red) showed both nuclear and cytosolic distribution. The nuclear envelope appeared to be normal across all regions of the hippocampus. Scale bar = 20 μm

We have previously established and characterized a FUS transgenic mouse model, in which transgene expressing wild-type (WT) and disease-linked mutation in FUS (R514G) were driven by the murine prion promoter, *prnp*-hFUS^WT^ and *prnp*-hFUS^R514G^, hereafter referred to WT-FUS and R514G-FUS (or simply R514G), respectively (Fig. 1a). These mice developed a progressive and mutant-enhanced phenotype and pathology resembling ALS [34]. As pathological FUS inclusions are also observed in the hippocampus of FTD patients [7, 8], we have previously shown that mice expressing wild-type human FUS at physiological levels developed progressive deficits in hippocampus-mediated cognition tests [49]. The data thus suggest an unappreciated aspect of hippocampal lesions due to FUS dysfunction. To further test this hypothesis, we focused our analysis on the hippocampus of R514G mice in this study (Fig. 1b).

Endogenous FUS was expressed predominantly in the NeuN-positive neurons within the hippocampus (left panel, Fig. 1c–e), as shown previously [49]. Similar to endogenous FUS, R514G transgene was found preferentially within the nucleus of NeuN-positive neurons throughout the hippocampus, including cornu ammonis 1 (CA1), CA3 and dentate gyrus (DG) (right panel, Fig. 1c–e). Furthermore, in contrast to the predominant nuclear localization of endogenous FUS (left panel, Fig. 1f–h), cytoplasmic as well as nuclear localization of R514G-FUS were observed across CA1, CA3, and DG regions of hippocampus without apparent FUS aggregates (right panel, Fig. 1f–h). Furthermore, RanGAP1 staining on the nuclear envelope showed normal morphology and was similar to to non-transgenic mice (Fig. 1f–h). The cytosolic localization of R514G-FUS was seen at 3 months of age and persisted to at least 12 months of age (Additional file 2: Fig. S1).

**Fig. 2:**
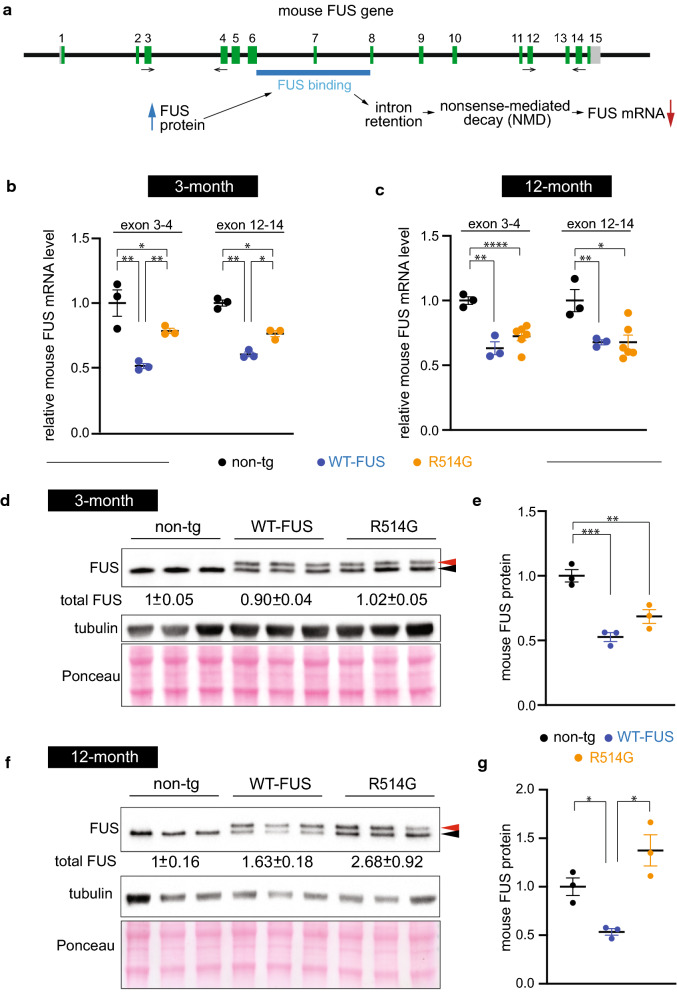
Age-dependent accumulation of endogenous FUS and ALS-linked mutant FUS. **a** Proposed model for FUS auto-regulation. FUS protein binds to the intron 6–7 of FUS mRNA, which results in retention of the introns. Retained introns contain premature stop codon that triggers nonsense-mediated decay to lower the FUS mRNA. Black arrow denotes the PCR primers for exon 3/4 and 12/14. **b**, **c** Relative mRNA expression of mouse FUS in the hippocampus of non-transgenic, wild-type FUS (WT-FUS) and R514G-FUS mice at 3 and 12 months of age. **b** Expression levels of mouse FUS mRNA in WT-FUS and R514G-FUS mice are approximately 51% and 78% to that of non-transgenic mice, respectively, at 3 months of age using exon 3/4 primers (n = 3, per genotype). Similarly, 60% and 76% of mouse FUS mRNA in WT-FUS and R514G-FUS mice using exon 12/14 primers. **c** Expression levels of mouse FUS mRNA in WT-FUS and R514G-FUS mice are approximately 63% and 72% to that of non-transgenic mice, respectively, at 12 months of age using exon 3/4 primers (n = 3, for non-transgenic and WT-FUS mice, n = 6 for R514G mice). Similarly, 68% and 68% of mouse FUS mRNA in WT-FUS and R514G-FUS mice using exon 12/14 primers. **d**–**g** Immunoblot of FUS and tubulin using total hippocampal lysates from 3 to 12 month old non-transgenic, WT-FUS and R514G-FUS mice. FUS was probed with a monoclonal antibody (clone 4H11), which recognizes human and mouse FUS with similar affinity. **d** Human FUS transgene (red arrow) expressed in a level similar to the endogenous FUS (blue arrow) and the total FUS level is comparable among the non-transgenic, WT-FUS and R514G-FUS mice at 3 months of age. Tubulin was used as a loading control. Ponceaus S staining was also used to ensure similar loading. **e** Quantification of mouse FUS proteins in non-transgenic, WT-FUS and R514G-FUS mice at 3 months of age. **f** Accumulation of endogenous FUS (red arrow) and R514G-FUS (blue arrow) transgenes at 12 months of age. Tubulin was used as a loading control. Ponceaus S staining was also used to ensure similar loading. **g** Quantification of mouse FUS proteins in non-transgenic, WT-FUS and R514G-FUS mice at 12 months of age. *p < 0.05; **p < 0.01; ***p < 0.001; ****p < 0.0001

We, and others, have previously shown that FUS autoregulates its own level by at least in part binding to its own RNA [29, 34, 63, 64]. When FUS protein levels are high, the bindings resulted in retained introns (intron 6–7) that subsequently led to nonsense-mediated decay (NMD) and consequently a downregulation of the FUS mRNA [63, 64] (Fig. 2a). To determine whether endogenous mouse FUS mRNA is reduced in WT-FUS and R514G-FUS mice, we used two primer sets: one before the retained intron 6–7 (primers are on exon 3 and 4) and one after the retained intron 6–7 (primers are on exon 12 and 14). The endogenous mouse FUS mRNA in the R514G-FUS mice was reduced to 78% (*p* < 0.001, exon 3–4) and 76% (*p* = 0.014, exon 12–14) when compared with non-transgenic (non-tg) mice at 3 months of age (Fig. 2b). Furthermore, the down-regulation of endogenous FUS mRNA were also observed at 12 months of age, where FUS mRNA in the R514G-FUS mice was reduced to 72% (*p* < 0.0001, exon 3–4) and 68% (*p* < 0.05, exon 12–14) (Fig. 2c). When compared to the WT-FUS transgenic mice [34, 49], R514G-FUS transgene did not reduce the endogenous FUS to the extent of wild-type human FUS (*p* < 0.01, exon 3–4; *p* < 0.05, exon 12–14) at 3 months of age (Fig. 2b). The level of reduction of mouse mRNA was comparable between WT-FUS and R514G mice at 12 months of age (Fig. 2c). This suggested that the autoregulatory mechanism is utilized to maintain the homeostatic FUS level in the hippocampus, although R514G-FUS appears to be less effective in reducing endogenous mouse FUS mRNA at the younger age.

To investigate the accumulated FUS protein level, we used a monoclonal antibody that recognizes human and mouse FUS protein with comparable affinity (clone 4H11) [29, 34] to estimate the amount of human transgene and endogenous FUS protein using total hippocampal lysates. Quantification revealed reduced accumulation of endogenous FUS protein in the presence of the human WT-FUS and R514G-FUS transgene at 3 months of age. Compared to non-transgenic mice, this led to an overall comparable level of total FUS proteins in the WT-FUS and R514G-FUS mice (0.9 ± 0.04 for WT-FUS, 1.02 ± 0.05 for R514G-FUS; Fig. 2d). Consistent with the qRT-PCR result, the mouse FUS protein was reduced to 52% (*p *< 0.001) and 68% (*p* < 0.01) in WT-FUS and R514G-FUS mice, respectively (Fig. 2e). Strikingly, total FUS accumulated at higher levels in both WT-FUS (1.63 ± 0.18, *p* < 0.05) and R514G-FUS (2.68 ± 0.92, *p* < 0.01) mice as compared to non-transgenic mice at 12 months of age (Fig. 2f). Furthermore, although endogenous mouse FUS accumulated at 50% of endogenous level in WT-FUS mice, mouse FUS were comparable to non-transgenic mice at 12 months of age (Fig. 2g). The data indicate that although FUS autoregulates its own level in the mouse central nervous system (CNS) to maintain a homeostatic level at 3 months of age, ALS-linked FUS mutation showed age-dependent increased accumulation by 12 months of age.

Collectively, the data suggest that: (i) R514G transgene showed similar expression pattern with the endogenous mouse FUS in the hippocampus; (ii) there were no apparent FUS aggregates and nuclear envelope defects in the hippocampus of R514G mice; (iii) total FUS protein level was initially maintained at the endogenous FUS expression level partly due to downregulation of mouse FUS mRNA; (iv) at the physiological expression level, R514G showed nuclear and cytosolic distribution; (v) although disease-linked mutation of FUS was not as effective as wild-type human FUS at 3 months of age, disease-linked mutation of FUS showed age-dependent protein accumulation, suggesting post-translational regulation of FUS protein may also be utilized for homeostatic FUS level.

### Reduction in spine density and deficits in long-term potentiation within the hippocampus of the R514G-FUS mice

**Fig. 3:**
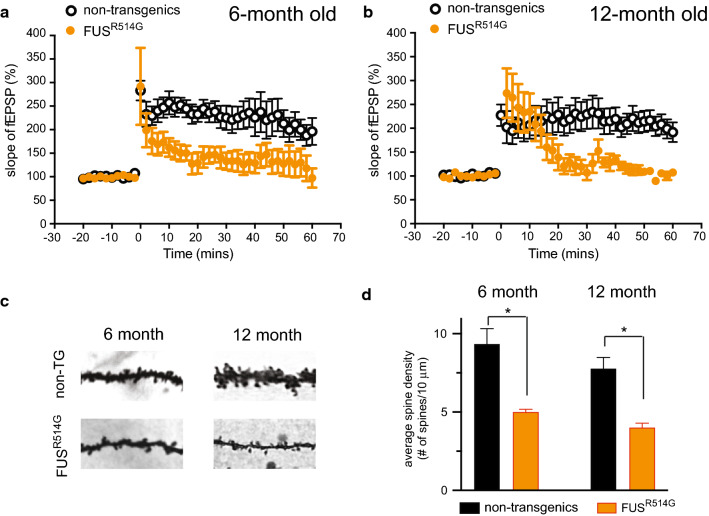
Reduction of long-term potentiation and spine density in the hippocampus of R514G mice. **a**, **b** LTP measurement of 6- and 12-month-old non-transgenic littermate control and R514G-FUS mice. fEPSPs were recorded in stratum radiatum (dendritic area) of the CA1 region following the stimulation of the Schaffer collateral pathway. LTP were induced with 4 tetanus stimulation delivered at 20-s intervals, each at 100 Hz for 1 s. Both 6- (**a**) and 12 months old (**b**) R514G-FUS mice showed reduced LTP when compared with their littermate controls. 6-month time-point: non-transgenic mice, n = 6; R514G-FUS mice n = 5. 12-month time-point: non-transgenic mice, n = 9; R514G-FUS mice n = 9. **c** Representative dendrite images of 6 month and 12 month old non-transgenic littermate control and R514G-FUS mice. Scale bar is 10 μm. **d** Quantification of spine density in 6 months and 12 months old non-transgenic littermate control and R514G-FUS mice. Spine density was reduced in the 6 and 12 months old R514G-FUS mice. Data are expressed as mean ± SEM; asterisks indicate significant differences between groups, n = 3 per genotype per time-point (*p < 0.05; unpaired t-test)

To determine whether there might be hippocampal lesions in R514G-FUS mice, we first examined whether there was any electrophysiological abnormality. Using a well-established long-term potentiation (LTP) paradigm, we recorded the field excitatory postsynaptic potential (fEPSP) from the CA1 dendritic field before and after the high frequency tetanic stimulation of Schaffer collateral/commissural pathway in the hippocampus isolated from 6- and 12-months old mice. LTP was measured after tetanic stimulation by four trains of 1-second 100 Hz stimulations. We saw a marked impairment in LTP in both 6- and 12-months old R514G-FUS mice as compared to controls (Fig. 3a, b).

To investigate whether LTP reduction in R514G-FUS mice is correlated with potential dendritic spine defects, we examined the spine density in the CA1 regions of hippocampus at 6 and 12-months old time points using Golgi-silver staining (Fig. 3c). Quantification on dendritic spines at the basal dendrite of CA1 pyramidal neurons showed 53% (*p* < 0.05) and 51% (*p* < 0.05) reduced spine density between non-transgenic littermate controls and R514G-FUS mice at 6- and 12-months of age, respectively (Fig. 3d). Therefore, R514G-FUS mice showed a reduction in spine density and long-term potentiation (LTP) in the hippocampus at 6 months and persist to 12 months of age. When compared with WT-FUS transgenic mice [49], these effects on synaptic plasticity and spine density appear to be enhanced by the ALS-linked R514G mutation, as there was a clear reduction of spine density and LTP as early as 6-month of age in R514G mice, but not WT-FUS mice.

### R514G-FUS transgenic mice show hippocampus-mediated cognitive deficits

**Fig. 4:**
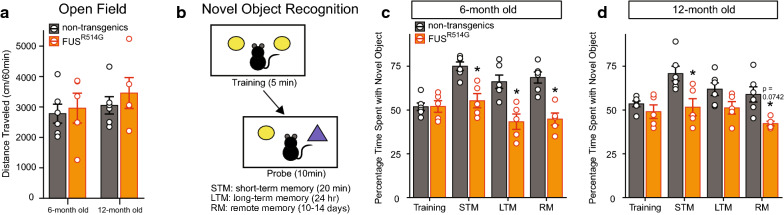
R514G-FUS animals show deficits in recognition memory. **a** R514G mice were assessed for locomotor activity in the open field test. No significant difference in exploratory behaviors were detected between non-transgenic and R514G mice. **b** A schematic showing the novel object recognition paradigm. **c**, **d** R514G mice showed significantly lower time spent with the novel object compared to non-transgenic mice at both 6 and 12 months old, for the short-term, long-term and remote memory probes (p = 0.072 for 12-month-old, remote memory probe; ps < 0.06 for all other probes). For all the tests shown here, there were 5 R514G mice and 6 non-transgenic mice. Orange circles and bars represent R514G mice, and black circles and grey bars represent non-transgenic mice

Since R514G mice developed motor deficits in gaits [34], we first assessed their exploratory behavior using the open field test for any potential locomotor deficits. The cumulative locomotor activity was analyzed using a two-way ANOVA across genotype and age. There were no significant main effects for genotype or age, suggesting that both non-transgenics and R514G-FUS mice showed similar exploration levels in the open field (Fig. 4a).

We assessed the recognition memory of the mice in the novel object recognition test, which in part, depends on hippocampus (Fig. 4b). The mixed-design ANOVA showed a significant main effect of genotype and test day, with a significant genotype by test day interaction (Fig. 4c, d; genotype: *F*_(1, 4669)_ = 70, *p *< 0.0001; test day: *F*_(1, 1674)_ = 8.31, *p *< 0.0001; and genotype by test day: *F*_(1, 1170)_ = 5.81, *p *=0.00130). There was no significant main effect of age. Pairwise comparisons showed that there were no significant differences for the percentage time spent with the novel object between R514G-FUS and non-transgenic mice during the training sessions at both 6 and 12 months of age. The short-term memory (STM) as tested 20 min after initial exposure, long-term memory (LTM) as tested 24 h later and remote memory (RM) as tested 10–14 days later probe tests, in 6 months old R514G mice showed significantly lower percentage of time spent with the novel object, compared to their non-transgenic counterparts (ps < 0.006). At 12 months of age, the probe tests showed R514G mice spending lower percentage of time with the novel object during the STM (*p *= 0.0190) and RM (*p* = 0.0742) probe tests, but did not reach significance level in RM, compared to their non-transgenic counterparts. The pairwise comparison for the LTM probe was not significantly different. Both genotypes spent similar amount of total interaction time with both objects during the testing sessions. The data suggests that R514G mice have deficits in recognition memory as early as 6 months of age and these deficits persisted to 12 months of age.

**Fig. 5:**
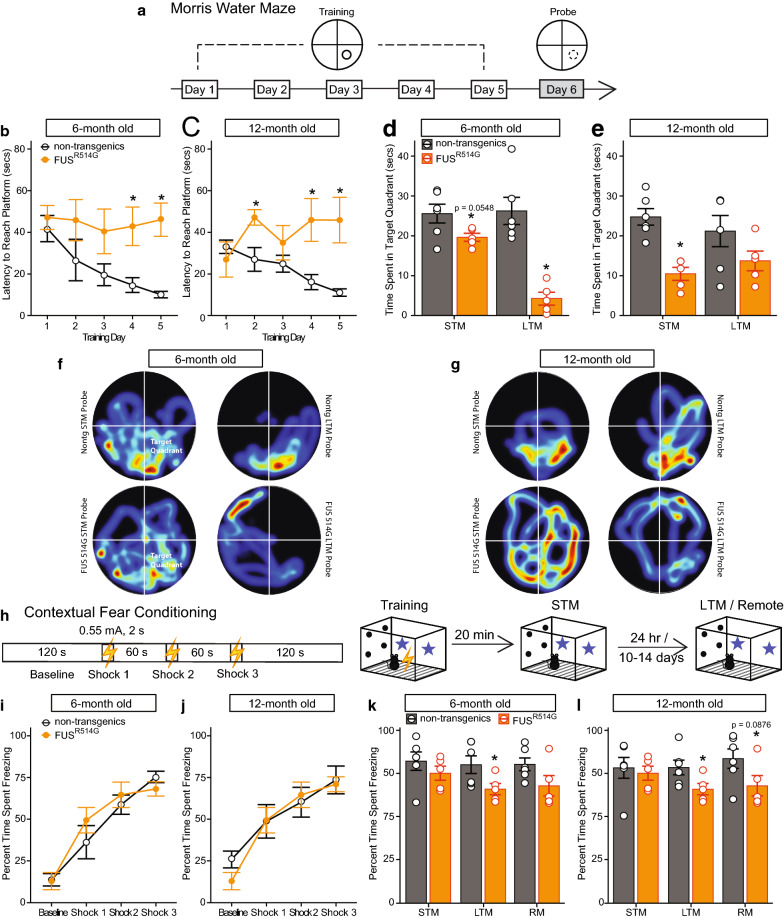
R514G-FUS animals show hippocampus-dependent cognitive deficits. **a** A schematic showing the spatial acquisition of the hidden platform in the Morris water maze paradigm. **b**, **c** The latencies to reach the hidden platform across five training days at 6 and 12 months. The latencies of R514G-FUS animals remained high and were significantly higher than non-transgenic animals on training days 4 and 5 (ps < 0.05). **d**, **e** Two probe tests were performed with the short-term memory probe carried out 20 min after the first reversal training session, and the long-term memory probe carried out 16–24 h after the fifth training session. R514G-FUS mice spent less time in the target quadrants during the STM and LTM probes compared to non-transgenic mice. **f**, **g** The average graphical representation for the search pattern of R514G-FUS and non-transgenic mice during each probe that was carried out at 6 and 12 months. **h** A schematic diagram showing the contextual fear conditioning paradigm that was used to assess hippocampal-dependent contextually acquired fear memory. **i**, **j** Percentages of freezing during training were recorded during the contextual fear conditioning paradigm. There were no significant differences in the percentages of time spent freezing between R514G-FUS and non-transgenic mice across both ages. **k**, **l** Percentages of freezing during probes were recorded during the contextual fear conditioning paradigm. R514G-FUS mice showed reduced freezing during the long-term and remote memory probes at both 6 and 12 months old. For all the tests shown here, there were 5 R514G-FUS mice and 6 non-transgenic mice. Orange circles and bars represent R514G-FUS mice, and black circles and grey bars represent non-transgenic mice

To further evaluate whether these mice developed hippocampus-mediated cognitive deficits, we performed two cognitive tasks requiring intact hippocampus [52, 65]. First, hippocampus-mediated spatial learning and memory was assessed using the Morris water maze fixed platform test paradigm. Mice were trained to find the hidden escape platform using distal cues placed around the maze for 5 days (Fig. 5a). The mixed-design ANOVA showed a significant main effect of genotype, and genotype by training day interaction (Fig. 5b, c; genotype: *F*_(1, 10703)_ = 41.6, *p *< 0.0001 and genotype by training day: *F*_(1, 4114)_ = 4.00, *p *=0.00493). Pairwise comparisons showed that R514G-FUS mice took significantly longer to reach the platform on training days 4 and 5, compared to their non-transgenic counterparts at both 6 and 12 months of age (*ps* < 0.05). The STM and LTM probe tests that were carried out to assess spatial memory showed a significant main effect of genotype, almost significant main effect of probe test, and a significant genotype by age by probe test (Fig. 5d–g; genotype: *F*_(1, 1686)_ = 44.9, *p *< 0.0001; probe test: *F*_(1, 154)_ = 4.11, *p *=0.0502; genotype by age by probe day: *F*_(1, 357)_ = 9.51, *p *=0.00391). Pairwise comparisons showed that R514G-FUS mice spent significantly less time in the target quadrant during the STM probe compared to their non-transgenic counterparts at 6 and 12 months of age (p = 0.0548 and p = 0.000449 respectively). During the LTM probe, R514G-FUS mice showed a significant reduction in time spent in the target quadrant compared to their non-transgenic counterparts at 6 months old (*p* = 0.000624), but not at 12 months old.

Hippocampus-mediated fear memory was assessed using the contextual fear conditioning test paradigm (Fig. 5h). The mixed-design ANOVA for percentage of freezing time during the training showed no significant main effect of genotype or age, but only for the number of shock exposures (Fig. 5i**–**l; *F*_(1,38771)_ = 47.7, *p *< 0.0001). STM, LTM and RM probe tests were performed to assess the retention of the contextual-dependent fear memory. The mixed-design ANOVA showed only a significant main effect of genotype (*F*_(1, 31913)_ = 14.5, *p *=0.000367). Pairwise comparisons showed that R514G-FUS mice froze significantly less during the LTM probe compared to their non-transgenic counterparts at both 6 and 12 months old (*ps* < 0.05). R514G-FUS mice also showed a lowered freezing time trend compared to non-transgenic mice at the remote probe, although this was not significant (*p* = 0.118 at 6 months old and p = 0.0876 at 12 months old). Taken together, the data suggests that R514G-FUS mice developed deficits in hippocampus-dependent cognition tasks as early as 6 months of age and the deficits persisted to 12 months of age.

### Transcriptomic analysis identified disrupted biological pathways for nonsense-mediated decay, protein homeostasis and mitochondrial functions in the hippocampus of R514G-FUS mice

**Fig. 6:**
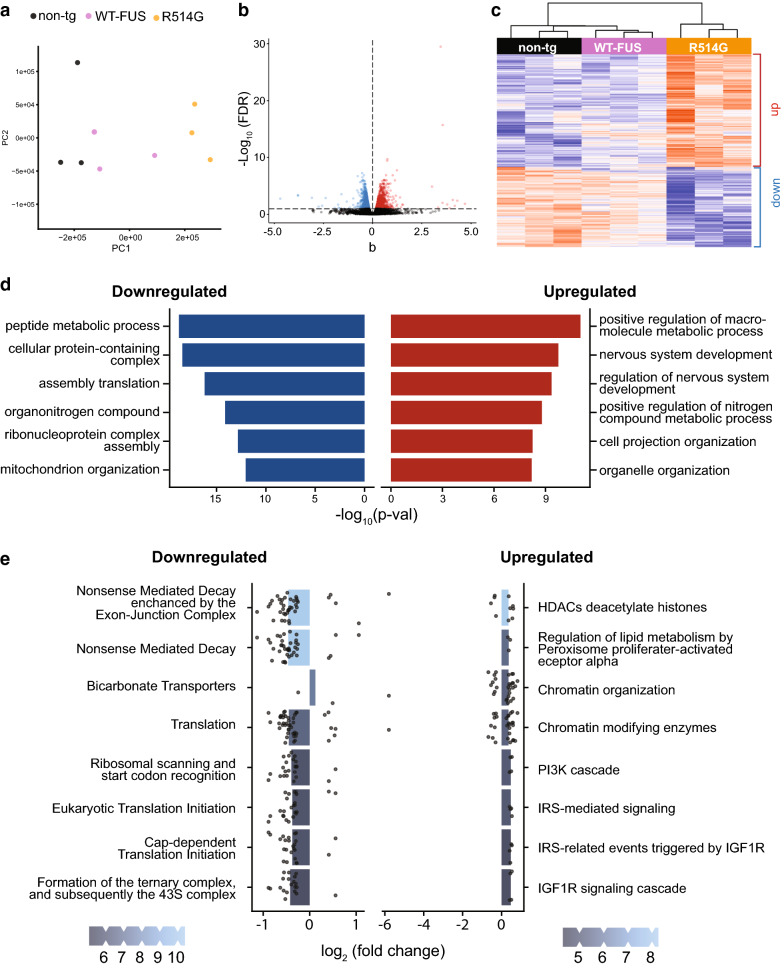
Transcriptomic analysis of hippocampus from R514G mice. PCA (**a**) and volcano (**b**) diagnostic plots for transcriptomic analysis of 12-month-old R514G-FUS mice, WT-FUS and non-transgenic controls showed changes in gene-expression. Red, blue and grey points represent upregulated genes, downregulated genes and genes below our significance threshold, respectively. **c** Heatmap of Z-centered log-scaled counts of differentially expressed genes across all samples. **d** The top overrepresented GO (Biological Process) terms in the up and down regulated differentially expressed genes in R51G mice as compared to non-transgenic mice. **e** The top enriched and depleted Reactome pathways resulting from gene set enrichment analysis. The height and color gradient of the bars reflect the normalized enrichment score and the log(p-value) of the Reactome pathway gene-sets in R514G-FUS mice, while the height of the points reflect the log2(foldchange) of the genes constituting the gene set in R514G-FUS mice. Majority of the genes in each gene-set are dysregulated in the same direction of the overall gene-set enrichment

To identify the potential molecular mechanisms underlying the hippocampus-mediated cognitive deficits, we performed RNA-Seq on the hippocampi isolated from 12 months old non-transgenic littermate controls, WT-FUS and R514G-FUS mice. Principal component analysis (PCA) clearly separated R514G-FUS mice from non-transgenic and WT-FUS mice (Fig. 6a). mRNA expression profiles in non-transgenic and R514G-FUS mice were distinct, with 1679 genes (785 downregulated genes, including FUS; 894 upregulated genes) showing statistically significant changes. R514G-FUS mice have 1992 significantly dysregulated genes as compared to WT-FUS mice. Among these, 74.6% of DEGs are commonly dysregulated in R514G mice as compared to both non-transgenic and WT-FUS mice (Fig. 6b, c). By contrast, only 23 genes showed significant dysregulation in WT-FUS compared to the non-transgenic mice, suggesting they are similar at the transcriptomic level (see also [49]). The large gene expression changes were consistent with cytosolic accumulation of R514G-FUS.

Gene ontology (GO) terms revealed functional divergences in the enriched up and downregulated GO terms (Fig. 6d). The top six downregulated GO terms were peptide metabolic process, cellular protein-containing complex, assembly translation, organonitrogen compound, ribonucleoprotein complex assembly and mitochondrion organization. The top 6 upregulated GO terms include positive regulation of macromolecular metabolic process, nervous system development, regulation of nervous system development, positive regulation of nitrogen compound metabolic process, cell projection organization and organelle organization.

Gene set enrichment analysis using the Reactome database yielded further details about the functional divergence (Fig. 6e): the most significantly downregulated (negatively enriched) pathways have to do with translation regulation such as nonsense-mediated decay (NMD), NMD enhanced exon-junction complex, ribosomal scanning and start codon recognition, eukaryotic translation initiation, cap-dependent translation initiation and formation of ternary complex and subsequently the 43 s complex. The log fold change of all genes present in the pathways was also plotted and the majority of the genes in each pathway have reduced expression in the R514G samples. The upregulated pathways with positive enrichment including HDACs deacetylase histones, regulation of lipid metabolism by PPAR, chromatin organization and modifying enzymes, PI3K cascade, IRS-mediated signaling and IGF1R signaling cascade.

**Fig. 7:**
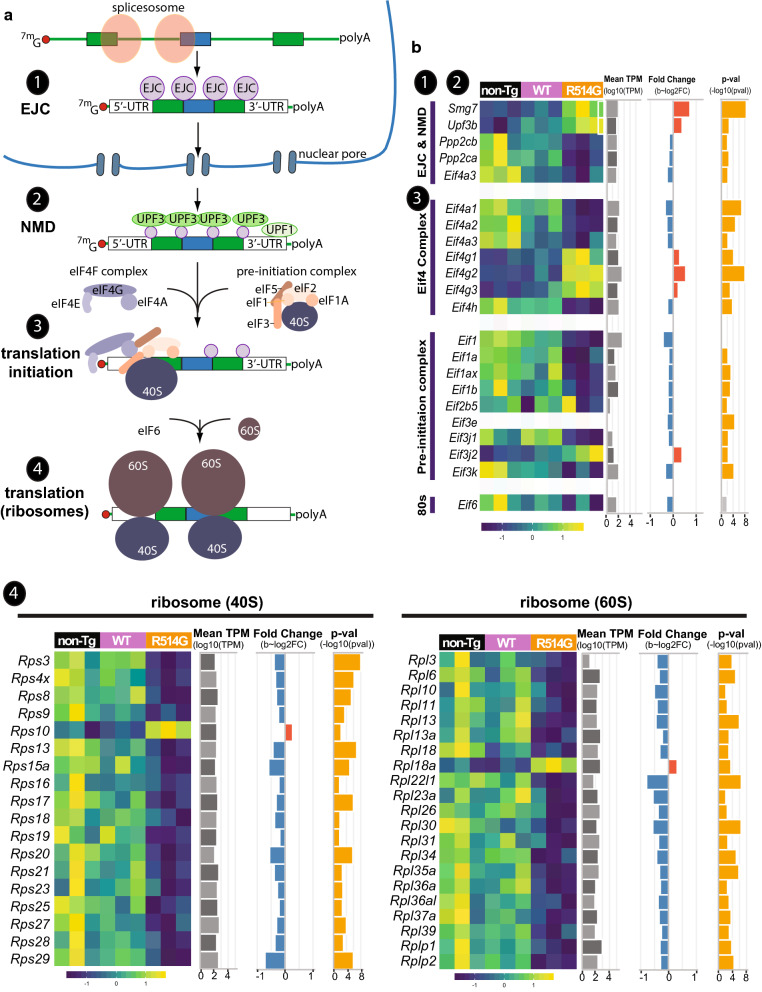
Disruption in nonsense-mediated decay (NMD) and protein translation in R514G mice. **a** Simplified schematic of mRNA processing to protein translation. Exon junction complexes (EJCs) are deposited at exon–exon junctions upon completion of splicing. Components of nonsense-mediated decay, such as UPF3, associate with EJCs. Translation initiation requires eIF4F complex that binds to the 5′-cap of mRNA and 43S pre-initiation complex containing 40S ribosome. **b** Heat map of the expression level of the DEGs for EJC-NMD, translation initiation and ribosomal subunits in non-transgenic, WT-FUS and R514G-FUS mice along with their mean expression level across all samples (log10(TPM)), and *p*-value (− log10(*p*-value)) and fold change (log2(fold change)) in R514G-FUS mice as compared to non-transgenic mice. Positive and negative fold change are colored red and blue respectively and *p*-values corresponding to significant *q*-values < 0.1 are colored in yellow

As the bioinformatic analysis identified the dysregulation of genes associated with exon-junction complexes (EJC), nonsense-mediated decay (NMD) and translation, we took a closer look at the DEGs in these pathway (Fig. 7). EJC binds to spliced exon–exon junctions and when there is ia stop codon located upstream of this spliced exon–exon junction, NMD is triggered. Among the components in EJC and NMD, eIF4a3, which binds directly to mRNA as part of EJC, was downregulated; whereas Upf3b, an activator of NMD [66], and SMG7, an adaptor for linking mRNA degradation machinery with Upf1 [67], were upregulated. In addition, reduction of protein phosphatase 2A (PP2A) (Fig. 7), which dephosphorylates Upf1 to reduce NMD activities [68], was also observed in the R514G-FUS mice. The data suggest that NMD may be enhanced in hippocampus of R514G-FUS mice.

The DEGs that are largely clustered in “translation” (Fig. 6e) can be roughly grouped into three categories: eIF4F complex, 43S pre-initiation complex and ribosomes (Fig. 7). The first two are involved in translation initiation, a key regulatory step in translation. eIF4F binds to the 5′-cap of mRNA to promote translation initiation and is composed of eIF4A, eIF4G and eIF4E (Fig. 7a). Among these, eIF4A1, eIF4A2, eIF4A3, and eIF4H were downregulated, whereas, eIF4G1, eIF4G2, and eIF4G3 were upregulated (Fig. 7b). The 43S preinitiation complex (43S PIC) is recruited by eIF4F and is composed of small ribosomal subunit (40S) bound by the initiation factors, eIF1, eIF1A, eIF2, eIF3, and eIF5 (Fig. 7a). Among these, eIF1, eIF1A, eIF1ax, eIF1b, eIF3j1, and eIF3k were downregulated (Fig. 7b). Strikingly, 49% of ribosomal proteins were downregulated (18 out of 33 in 40S subunits; 22 out of 49 in 60S subunit). The data suggest a potential impairment in protein translation.

**Fig. 8:**
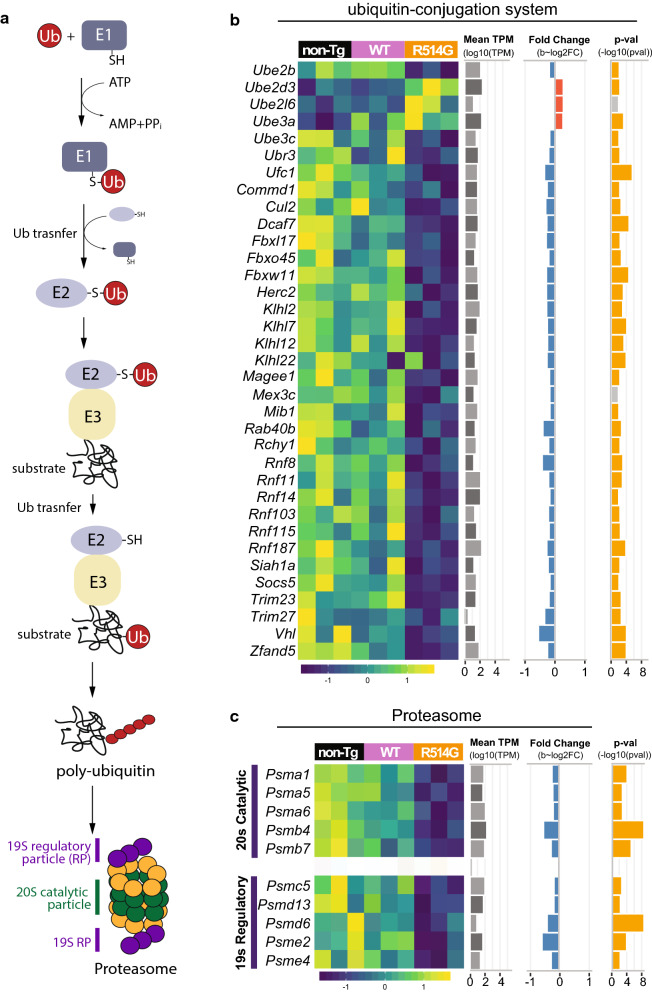
Defected ubiquitin–proteasome axis in R514G mice. **a** Simplified schematic of ubiquitin–proteasome pathway. Ubiquitin is attached to the substrate via E1, E2 and E3. The poly-ubiquitinated proteins were targeted to proteasome for degradation. Proteasome is composed of 20S catalytic particle and 19S regulatory particle (RP). **b** Heat map of the expression level of the DEGs for ubiquitin–proteasome pathway in non-transgenic, WT-FUS and R514G-FUS mice along with their mean expression level across all samples (log10(TPM)), and *p*-value (− log10(*p*-value)) and fold change (log2(fold change)) in R514G-FUS mice as compared to non-transgenic mice. Positive and negative fold changes are colored red and blue respectively and *p*-values corresponding to significant *q*-values < 0.1 are colored in yellow

Intriguingly, we also uncovered that many of the downregulated DEGs were mapped to protein ubiquitination and the proteasome pathway (Fig. 8). The addition of ubiquitin to its substrate requires 3 steps: activation, conjugation and ligation, which are performed by ubiquitin-activation enzymes (E1s), ubiquitin-conjugation enzymes (E2s) and ubiquitin ligases (E3s). The resulting polyubiquitinated proteins are targeted to proteasomes for degradation (Fig. 8a). We found 4 E2s and 33 E3s were downregulated in the hippocampus of R514G mice. Furthermore, 8 out of 19 (42.1%) in 20S catalytic particle and 5 out of 18 (27.8%) in 19S regulatory particle in proteasome were downregulated. The data suggest a potential impairment in proteasome-dependent degradation.

**Fig. 9:**
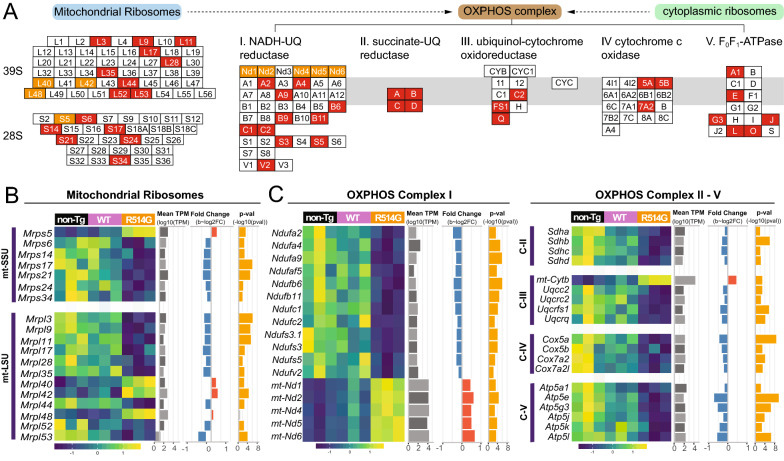
Defected mitochondrial ribosome-OXPHOS complexes in R514G mice. **a** Simplified schematic of mitochondrial ribosomes and OXPHOS complexes. Components of OXPHOS complexes were translated by mitochondria and cytosolic ribosomes. **b** Heat map of the expression level of the DEGs for mitochondrial ribosomes and OXPHOS complexes in non-transgenic, WT-FUS and R514G-FUS mice along with their mean expression level across all samples (log10(TPM)), and *p*-value (− log10(*p*-value)) and fold change (log2(fold change)) in R514G-FUS mice as compared to non-transgenic mice. Positive and negative fold changes are colored red and blue respectively and *p*-values corresponding to significant *q*-values < 0.1 are colored in yellow

Similar to cytosolic ribosomes, we observed ~ 25% of mRNAs encoding mitochondrial ribosomes were selectively deregulated in the hippocampus of R514G-FUS mice. These included 8 out of 31 subunits in 28S (25.8%), 12 out of 48 subunits in 38S (25%). Among them, 80% of mitochondrial ribosomal proteins were downregulated (7 and 9 were downregulated in 28S and 38S, respectively), suggesting potential deficits in mitochondrial translation. Since mitochondrial translation is required for a few subunits of the oxidative phosphorylation (OXPHOS) complexes, we extended our analysis to the four respiratory enzyme complexes (complex I-IV) and ATP synthase (complex V). 11 out of 42 in complex I NADH-UQ reductase, all 4 subunits in complex II succinate-UQ reductase, 3 out of 9 in complex III, ubiquinol-cytochrome oxidoreductase, 3 out of 17 in complex IV, cytochrome c oxidase, and 6 out of 16 in complex V, F0F1-ATPase were downregulated. It is worth mentioning that 5 of 6 mitochondrial genome-encoded complex I subunits (mt-Nd1, mt-Nd2, mt-Nd4, mt-Nd5 and mt-Nd6) were upregulated. The data suggest that there are potential mitochondrial dysfunctions in the hippocampus of R514G mice.

### Widespread brain volume and functional connectivity changes in R514G mice

**Fig. 10:**
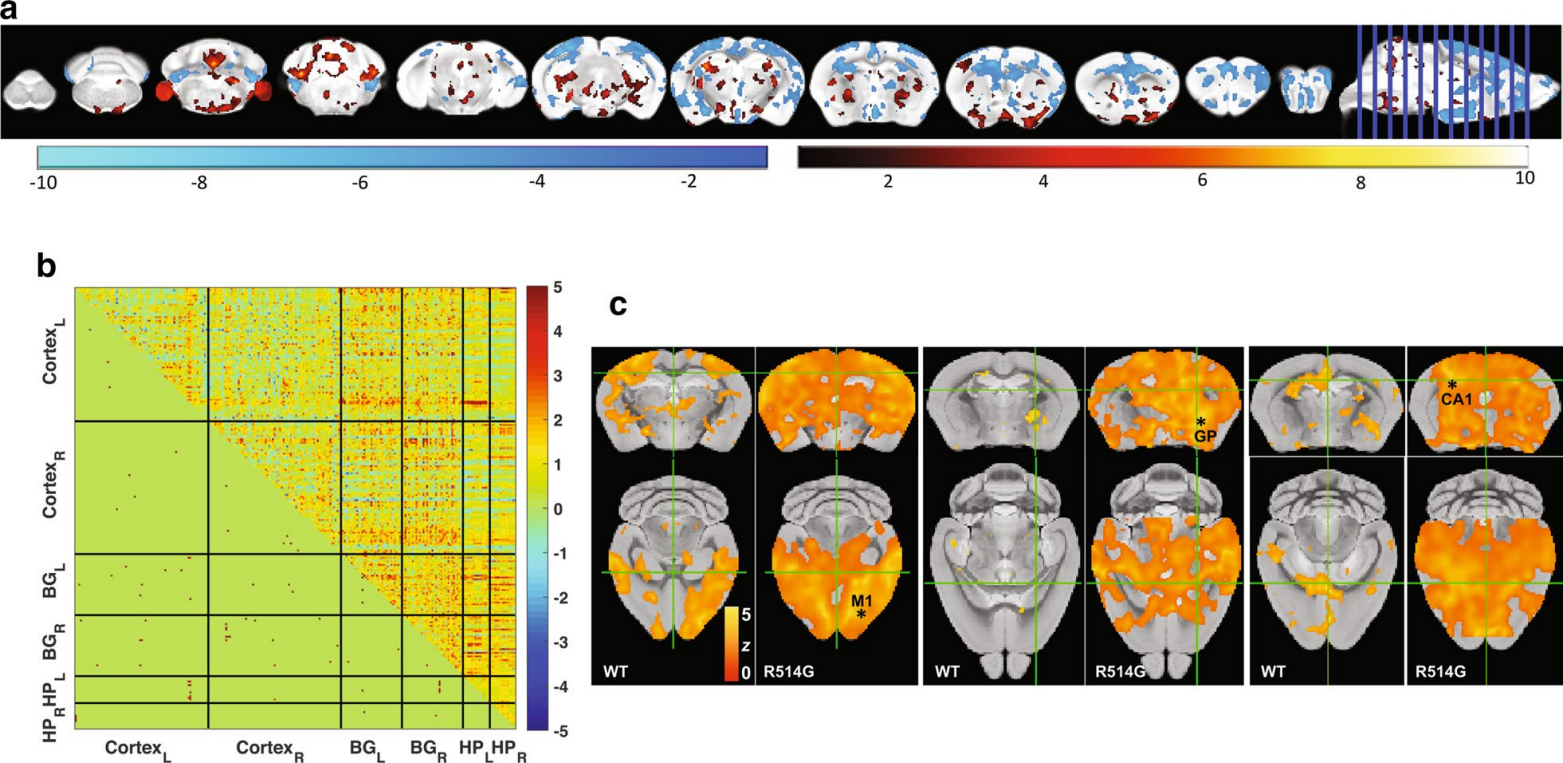
Wide-spread brain volume changes and functional connectivity changes in R514G-FUS mice. **a** Brain structural changes between R514G and the control detected by tensor-based morphometry (*p *< 0.01, threshold-free cluster enhancement corrected). Wide-spread atrophy (cold color) can be found in the motor, insular, medial prefrontal, piriform, somatosensory and visual cortices and amygdala. Localized brain volume increase (hot color) can be seen mostly in subcortical areas, including ventral pallidum, lateral hypothalamus. Hippocampal CA3, medial geniculate nucleus and paraflocculus. N = 5 per genotype. **b** Functional connectivity measured by resting-state fMRI. (C) Connectivity matrix of resting-state functional connectivity change between R514G and the control (*p* < 0.001 uncorrected). Significant connectivity enhancement was seen between the left hippocampal CA2/3 and left visual cortex, and between the right hippocampal CA2/3 and the left diagonal band (n = 4 for non-transgenic, n = 5 for R514G-FUS mice)

Given that multiple important biological pathways were affected in the hippocampus of R514G-FUS mice, we suspected that the whole CNS may be affected. To this end, we performed non-invasive brain imaging. Voxel-wise morphometric analysis from the structural MRI revealed that R514G-FUS mice exhibited widespread reduction in cortical and subcortical volumes when compared to non-transgenic mice, in areas such as the motor, somatosensory, visual, cingulate and retrosplenial cortices, the hippocampus and amygdala (Fig. 10a). There was also increased brain volume in the piriform cortex, basal ganglia and cerebellum of R514G-FUS relative to non-transgenic mice. Further analysis indicated that there were no significant changes in total brain volume (mean ± SD = 346.6 ± 4.7 mm^3^ for R514G-FUS and 346.0 ± 3.4 mm^3^ for non-transgenic). Regional analysis also showed that R514-FUSG mice had significantly larger globus pallidus volume compared to non-transgenic mice (1.90 ± 0.027 mm^3^ for R514G vs 1.75 ± 0.066 mm^3^ for non-transgenic; *p *< 0.005).

To further investigate of the network-level pathophysiology in the CNS of R514G-FUS mice, resting-state functional MRI, which measures functional connectivity of the brain through correlated oscillation in the blood oxygenation level dependent signal among brain regions (for review, see [69, 70]), was utilized. Brain-wide functional connectivity analysis of the resting-state fMRI revealed that the R514G-FUS mice have increased connectivity mostly in the subcortical-cortical connections and scattered corticocortical connections relative to non-transgenic mice (Fig. 10b). Further voxel-wise analysis showed that the motor cortex connectivity expanded from sensorimotor areas in non-transgenic mice, to the entire neocortex and thalamus bilaterally in R514G-FUS mice (Fig. 10c). The globus pallidum and dorsal hippocampus also showed enhanced connectivity in R514G-FUS mice to distributed cortical areas particularly the sensorimotor areas (Fig. 10c).

## Discussion

In this study, we investigated how ALS-linked mutation (R514G) in FUS may cause hippocampal dysfunction. Similar to endogenous FUS, the R514G-FUS transgene is largely restricted to hippocampal neurons. At the physiological expression level, R514G showed nuclear and cytosolic localization, indicating the disruption of nuclear localization due to the mutation in NLS. No apparent nuclear envelope defects and no FUS aggregates were observed. At 3 months of age, FUS proteins were maintained at a homeostatic level, at least in part, by downregulating the endogenous mouse FUS mRNAs. Despite endogenous mouse FUS mRNAs remaining downregulated at 12 months of age, R514G-FUS and the endogenous FUS protein accumulated to a higher level compared to the non-transgenic mice. The data suggest that the age-dependent protein accumulation occurs post-translationally. These R514G-FUS mice develop deficits in hippocampus-mediated cognition tasks, such as the Morris water maze and contextual fear conditioning, as early as 6 months of age, and the deficits persisted through to 12 months of age. These behavioral deficits were accompanied by the reduction in spine density of CA1 pyramidal neurons and LTP at the Schaffer collateral/commissural-CA1 pathway, suggesting that ALS-linked FUS mutation triggers hippocampal dysfunctions. Unbiased transcriptomic analysis revealed the deregulated genes clustered in the nonsense-mediated decay (NMD)-translation axis, ubiquitin–proteasome pathway and OXPHOS system in the mitochondria. The data suggest a potentially enhanced NMD, reduced protein translation, defective protein degradation and deficient OXPHOS in the hippocampus of R514G-FUS mice. Among these, defective protein degradation may account for the age-dependent accumulation of R514G-FUS and mouse FUS proteins. Lastly, using in vivo imaging techniques, we found that R514G-FUS mice exhibited widespread reduction in cortical volumes but enhanced functional connectivity between hippocampus, basal ganglia and neocortex. Taken together, our data indicate that at the physiological level, the ALS-linked mutation in FUS is able to cause hippocampal dysfunction, likely via affecting protein homeostasis and mitochondrial dysfunctions.

We have previously analyzed WT-FUS transgenic and found that WT-FUS transgenic mice developed cognitive deficits accompanied by age-dependent reduction in spine density and long-term potentiation (LTP) [49]. The effects appear to be enhanced by the ALS-linked R514G mutation, as there was evidences of spine density reduction and altered LTP as early as 6-month of age in R514G mice, but not WT-FUS mice. At the transcriptomic level, only a small subset of gene changes was identified [49]. By contrast, we found multiple pathways, such as nonsense-mediated decay, protein homeostasis and mitochondrial functions, were affected in the R514G mice when compared with non-transgenic and WT-FUS mice. Given that WT-FUS and R514G-FUS transgenes expression mimics the endogenous FUS pattern, the data collectively suggest that ALS-linked mutation in FUS may incur toxic gain-of-functions via affecting multiple biological processes in the hippocampus. Future studies will be needed to investigate whether similar alternation also occurs in other neurons and/or other brain regions, such as prefrontal cortex and spinal cord.

Maintaining homeostatic FUS levels appears to be essential for CNS health. Overexpression of wild-type human FUS in the CNS of mice is sufficient to trigger neurological phenotypes and early lethality [34, 71]. Patients with mutations in the 3′-UTR of FUS have higher accumulated FUS levels and develop ALS [72]. Furthermore, three different FUS mouse models: two knock-in models [38, 73] and one BAC transgene model [29] showed that total FUS accumulated at a level similar to endogenous FUS. At least two mRNA-mediated mechanisms have been proposed to maintain homeostatic FUS level: the regulatory loop of (i) FUS protein-FUS mRNA, and (ii) FUS-microRNA (miRNA) interaction. In the FUS protein-FUS mRNA regulatory loop, FUS proteins bind to intron 6–7 of FUS mRNA. Elevated FUS proteins cause increased binding to FUS mRNAs leading to the retention of intron 6–7. This intron retention can either result in a pre-mature stop codon, rendering the mRNAs as a substrate for NMD, thereby lowering FUS mRNA levels [63], or alternatively leading to nuclear retention of FUS mRNA, thereby lowering available FUS mRNA for translation [64]. In the FUS-miRNA regulatory loop, FUS mRNAs are targets of miR-141/200a. When FUS protein levels are high, FUS facilitates the expression of miR-141/200a, which, in turn, inhibits FUS protein production [74]. ALS-linked mutations within the nuclear localization signal (NLS) of FUS have been shown to reduce binding to intron 6–7 of FUS mRNA [64], which presumably would lead to higher accumulation of FUS mRNA available for translation. Consistent with its nuclear and cytosolic distribution, R514G-FUS appeared to retain some of its ability to autoregulate mouse FUS mRNA at both 3 and 12 months of age. It is worth mentioning that the ability of R514G-FUS to downregulate mouse FUS mRNA was not as effective as wild-type human FUS transgene at both 3 and 12 months of age. At the protein level, total FUS comprising of human R514G-FUS and mouse FUS were comparable to mouse FUS of the non-transgenic mice at 3 months of age consistent with qRT-PCR results. However, both human R514G FUS and mouse FUS showed elevated accumulation at 12 months of age, despite mouse FUS mRNA remaining downregulated to a level similar to that at 3 months of age. Thus, the data suggest that post-translational mechanisms may be used to maintain the homeostatic FUS protein level. Indeed, unbiased transcriptomic analysis revealed a reduction in ubiquitin–proteasome pathway in R514G mice, suggesting that the impaired protein degradation may account for the elevated FUS accumulation.

Increased Upf3b and SMG7 and reduced PP2A suggest a potentially enhanced NMD in R514G-FUS mice. This is consistent with a recent report showing enhanced NMD in ALS-linked FUS mutations using in vitro cell culture system [75]. Our transcriptomic analysis also revealed a reduction in components of eIF4F complex and 43S pre-initiation complex, suggesting impaired protein translation. This was further supported by the transcriptomic data that showed a reduction of 49% of mRNAs encoding ribosomal subunits. Similarly, about 25% of mRNAs encoding mitochondrial ribosomal proteins were reduced in R514G-FUS mice, suggesting potential damage to mitochondrial translation. Indeed, many components in the OXPHOS complexes that are required for oxidation-phosphorylation-mediated generation of ATP within mitochondria were reduced. Thus, it is likely that there are mitochondrial dysfunctions in R514G-FUS mice. This is consistent with the findings from the Wu and Manley laboratories. Wu laboratory showed that FUS directly interacts with mitochondrial chaperon HSP60 [76] and ATP5B [77] to affect mitochondrial functions. Whereas the Manley laboratory showed that FUS regulates the expression of OXPHOS components by directly bindings to the mRNAs encoding these proteins [48]. Our data are consistent with a progressive reduction of cytosolic ribosomes, proteasome and mitochondrial proteins that were observed in a knock-in FUS mouse model where exon 14 was deleted [73]. In addition, FUS protein carrying an aggressive mutation (P525L) has been shown to interact with energy metabolism and protein degradation pathway [78]. Taken together, our data indicate that defects in protein homeostasis and mitochondrial dysfunctions may contribute to hippocampal dysfunctions.

Noninvasive neuroimaging offers great potential for identifying translatable clinical biomarkers as it can be conducted in both mouse models and humans. Here, we explored the use of MRI for brain structure and connectivity and noted a widespread brain atrophy. For the small subset of human FTD-FUS cases, the clinical MRI signature generally display region-specific atrophy in the frontal and temporal cortices [79–81]. In particular, tissue atrophy in the caudate has also been consistently reported in several studies and is considered as a defining imaging signature to distinguish between FTD-FUS patients and different FTD pathologies [82, 83]. Caudate volume ratio has been reported to be higher in comparison to frontal volume. This further supports caudate atrophy to be disproportional in comparison to other brain regions in FTD-FUS individuals [84]. We found that R514G mice have significantly larger globus pallidus, a region highly innervated by the caudate. Together, these suggest abnormal basal ganglia may be affected by ALS-FUS mutation.

Furthermore, we observed reduced brain volume but increased functional connectivity in the R514G-FUS mice. This seemingly paradoxical result has been observed in clinical studies, where wide-spread brain atrophy accompanied increased resting-state functional connectivity [85, 86]. Compensation of neural loss and loss of local inhibitory circuitry has been proposed to explained these clinical observations [87]. In addition, we found the brain atrophy and functional connectivity changes extend beyond the motor pathways to various regions essential for learning and memory. The reduced hippocampal spine density and LTP could support the compensatory role of increased hippocampal functional connectivity. Alternatively, abnormal functional connectivity may reflect altered neural synchrony due to impaired LTP [88]. Although exact mechanisms remain to be delineated, our MRI findings suggest these imaging readouts could be potential biomarkers.

## Conclusions

Here, we showed that a disease-linked FUS mutation autoregulates its own protein and mRNA level in young adult mice but FUS protein accumulated at higher levels in the hippocampus during aging. Mice expressing disease-linked FUS mutation developed hippocampus-mediated cognitive deficits accompanied by (1) reduced spine density and long-term potentiation (LTP), and (2) disruptions in nonsense-mediated decay (NMD), protein homeostasis and mitochondrial functions at the transcriptomic level, and (3) widespread brain volume and functional connectivity changes. Taken together, our findings suggest that disease-linked mutation in FUS may lead to changes in proteostasis and mitochondrial dysfunction, which in turn affect brain structure and connectivity resulting in cognitive deficits. Therefore, our R514G mouse model presents broader neuronal damages and may be a good model to study dementia-related diseases.

## Supplementary Information

**Additional file 1: Table S1.** RT-PCR primers used in this study.

**Additional file 2: Fig S1.** Nuclear and cytosolic R514G-FUS localization in the hippocampus of 12-month-old *prnp*-FUS^R514G^ mice. (A-C) Confocal images of the CA1 (A), CA3 (B), and DG (C) regions that were co-labeled with endogenous FUS (magenta) or R514G-FUS transgene (red) and nuclear envelope marker, RanGAP1 (green), from non-transgenic and R514G-FUS mice at 12 months of age. Endogenous FUS (magenta) is restricted to nuclei, whereas R514G-FUS transgene (red) showed both nuclear and cytosolic distribution. The nuclear envelop appeared to be normal across all regions of hippocampus. Scale bar = 20 μm. Three biological replicates per genotype.

## Data Availability

All data generated or analysed during this study are included in this manuscript (and its supplementary information files). All original data of this study are available from the corresponding author upon request. RNA-seq data have been deposited in NCBI’s Gene Expression Omnibus with the GEO series accession number GSE157713. (https://www.ncbi.nlm.nih.gov/geo/query/acc.cgi?acc=GSE157713).

## Abbreviations

ALS: Amyotrophic lateral sclerosis
FTD: Frontotemporal dementia
FTLD: Frontotemporal lobar degeneration
FUS: Fused in sarcoma
NMD: Nonsense-mediated decay
OXPHOS: Oxidation phosphorylation
fMRI: Functional magnetic resonance imaging
LTP: Long-term potentiation
CNS: Central nervous system
NLS: Nuclear localization signal
RNAP II: RNA polymerase II
RNP: Ribonucleoprotein
AMPA: α-Amino-3-hydroxy-5-methyl-4-isoxazolepropionic acid
SynGAP1: Synaptic Ras GTPase-activating protein 1
CA: Cornu ammonis
DG: Dentate gyrus
fEPSP: Field excitatory postsynaptic potential
STM: Short-term memory
LTM: Long-term memory
RM: Remote memory
PCA: Principal component analysis
GO: Gene ontology (GO)
EJC: Exon-junction complex
