# Supplementary material for: Dysfunction in nonsense-mediated decay, protein homeostasis, mitochondrial function, and brain connectivity in ALS-FUS mice with cognitive deficits
Source: Acta Neuropathol Commun. 2021 Jan 6;9:9. doi: 10.1186/s40478-020-01111-4 (PMC7789430; doi:10.1186/s40478-020-01111-4)
Supplement: Supplementary file 1 — Additional file 1: Table S1. RT-PCR primers used in this study. [file 40478_2020_1111_MOESM1_ESM.docx]

**Table S1. RT-PCR primers used in this study**

| **Gene** | **Forward (5’->3’)** | **Reverse (5’->3’)** | **Comments** |
| --- | --- | --- | --- |
| GAPDH | catggccttccgtgttccta | cctgcttcaccaccttcttgat | Reference gene |
| HPRT | gctcgagatgtcatgaaggagat | aaagaacttatagccccccttga | Reference gene |
| ARHGDIA | ctcggggcagttacaacatcaagtc | gtcgccctgcccgtctcc | Reference gene |
| FUS | caacgagctggagactggaa | gccacgtcgatcatctccat | Exon 12 & Exon 14, mouse |
| FUS | ggccagtcagctgacacttcag | tgctgccatagcctccagtggaa | Exon 3 & Exon 4, mouse |
