# Supplementary figures and images for: Dysfunction in nonsense-mediated decay, protein homeostasis, mitochondrial function, and brain connectivity in ALS-FUS mice with cognitive deficits
Source: Acta Neuropathol Commun. 2021 Jan 6;9:9. doi: 10.1186/s40478-020-01111-4 (PMC7789430; doi:10.1186/s40478-020-01111-4)

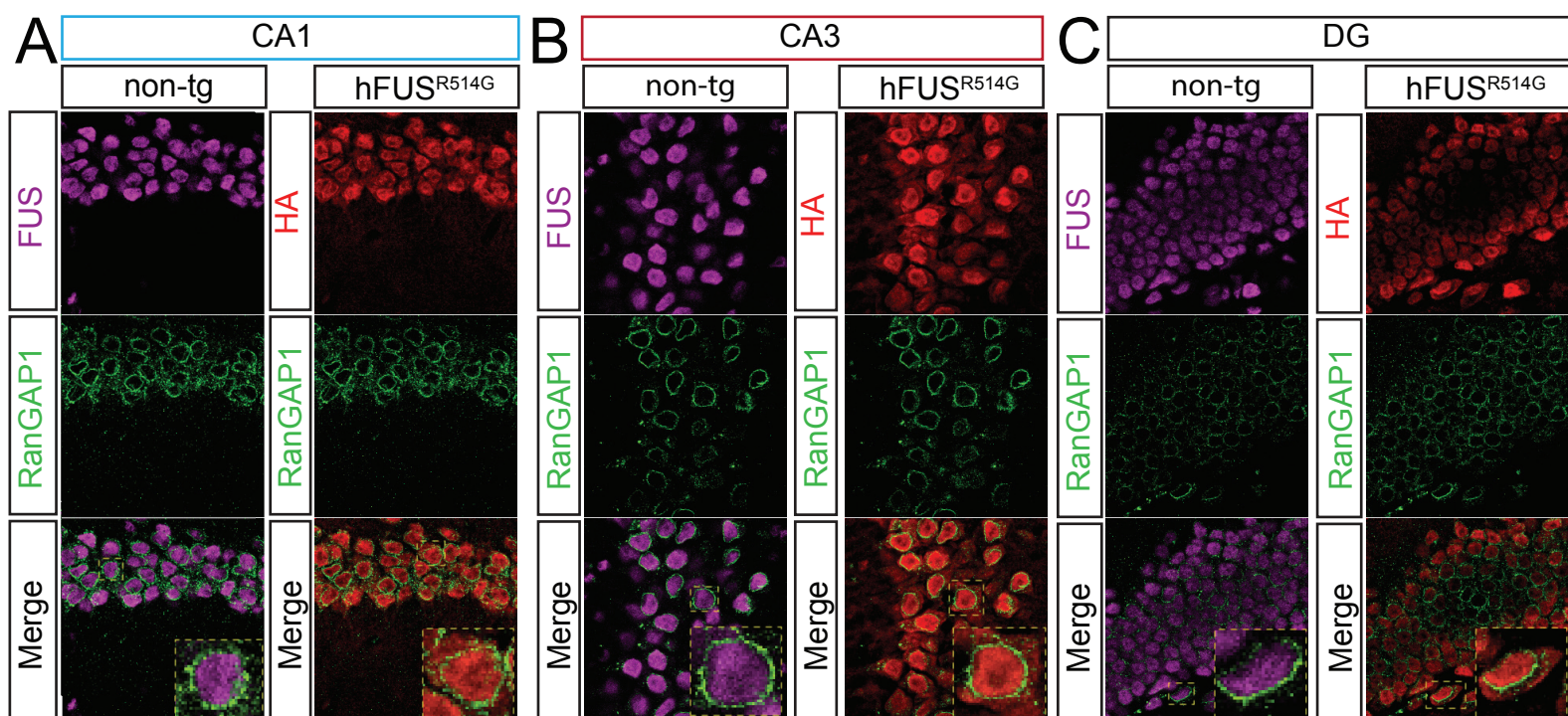

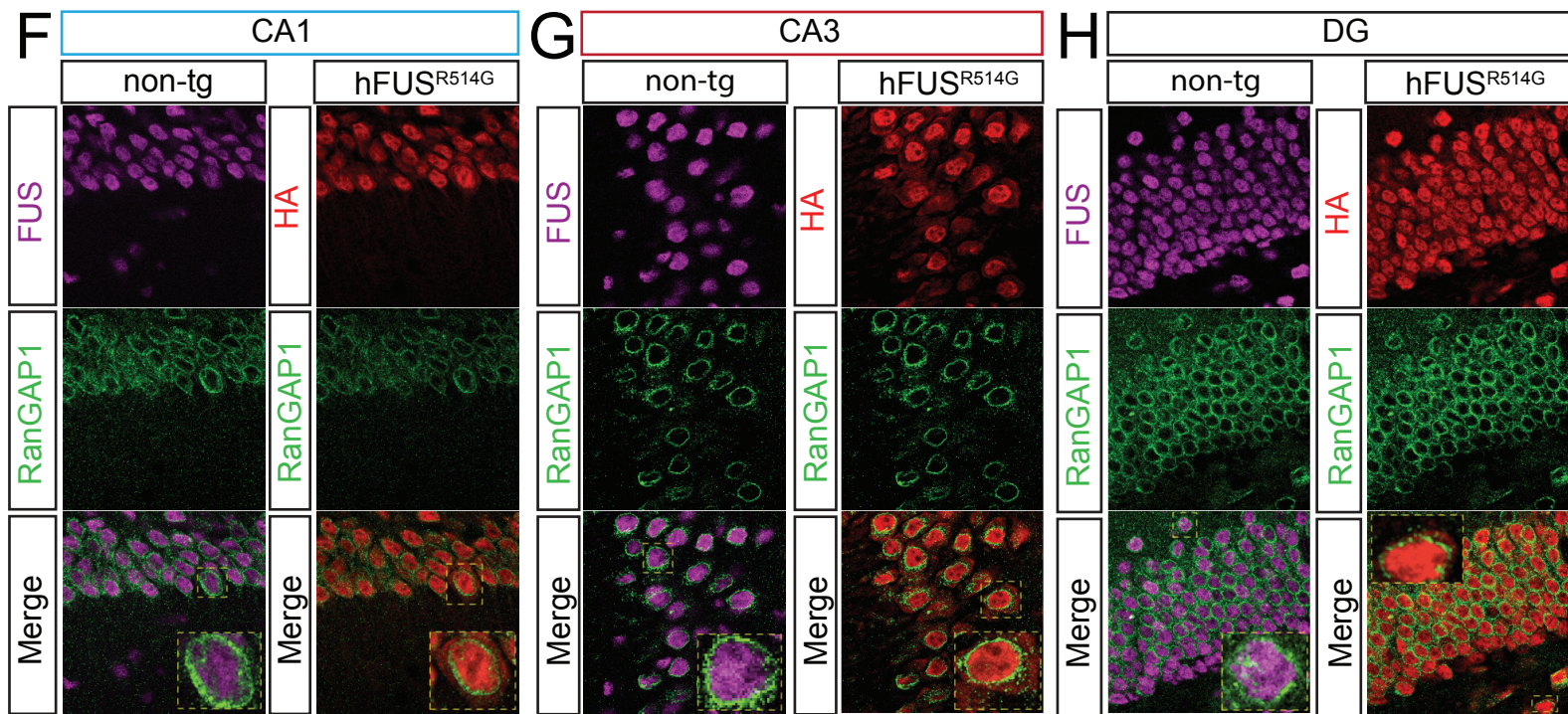

Supplement: Supplementary file 2 — Additional file 2: Fig S1. Nuclear and cytosolic R514G-FUS localization in the hippocampus of 12-month-old prnp-FUSR514G mice. (A-C) Confocal images of the CA1 (A), CA3 (B), and DG (C) regions that were co-labeled with endogenous FUS (magenta) or R514G-FUS transgene (red) and nuclear envelope marker, RanGAP1 (green), from non-transgenic and R514G-FUS mice at 12 months of age. Endogenous FUS (magenta) is restricted to nuclei, whereas R514G-FUS transgene (red) showed both nuclear and cytosolic distribution. The nuclear envelop appeared to be normal across all regions of hippocampus. Scale bar = 20 μm. Three biological replicates per genotype. [file 40478_2020_1111_MOESM2_ESM.pdf]
